# P03.10. The quality of integrative postgraduate medical education within the public health care system of Germany: the example of anthroposophic hospitals

**DOI:** 10.1186/1472-6882-12-S1-P263

**Published:** 2012-06-12

**Authors:** H Peter, S Eberhard, M Siegrist, P Orlow

**Affiliations:** 1 University of Witten/Herdecke, Herdecke, Germany; 2 Swiss Federal Institute of Technology, Zürich, Switzerland

## Purpose

Integrative medicine (IM) health care requires appropriate education in IM. In Germany, hospitals providing conventional medicine (CM) and anthroposophic medicine (AM) have been training residents in IM for over four decades. In view of growing public interest for IM, we evaluated the quality of postgraduate medical education (QPME) in these hospitals.

## Methods

We conducted an anonymous survey among all 215 residents of the 34 departments of all 11 German hospitals providing AM and IM and regular postgraduate medical education (PME), using an instrument with 71 scaled questions developed by the ETHZ for assessing QPME in CM hospitals, complemented by 22 additional questions for AM aspects of PME. Forty-two percent responded. Data were analyzed descriptively and according to department sizes and clinical disciplines.

## Results

QPME received the highest ratings in small (1-3 residents) departments, intermediate ratings in intermediate size departments (4-10 residents), and the lowest ratings in large (>11 residents) departments. This was consistent for overall satisfaction in CM and AM, overall clinical competency in CM and AM, cultures in learning, leadership, decision making, organization, evidence based medicine, and for the working situation, although working hours were comparable. Among the clinical disciplines, internal medicine, pediatrics, surgery and anesthesiology scored lower than gynecology/obstetrics, psychiatry/psychotherapy, and neurosurgery, whereby working hours were high in surgery and internal medicine, but also in neurosurgery, and low in pediatrics and psychiatry/psychotherapy. Reasons for constraints to an optimal QPME in AM were given as (in this order): high work load, too much administrative work, organizational problems; inadequate payment, not enough relation to practice, insufficient AM competency of educators, lacking financial resources, and insufficient didactic competencies of educators were not very important reasons for constraints to an optimal QPME.

## Conclusion

QPME in IM was related to department sizes and disciplines. Main reasons were working loads (not working hours) and organizational problems; less important reasons were inadequate payment or insufficient clinical or didactic competencies of educators.

